# Spiral Enteroscopy Utilizing Capsule Location Index for Achieving High Diagnostic and Therapeutic Yield

**DOI:** 10.1155/2015/793516

**Published:** 2015-11-19

**Authors:** Rohan Mandaliya, Jason Korenblit, Brendan O'Hare, Anastasia Shnitser, Ramalinga Kedika, Rebecca Matro, Dina Halegoua-De Marzio, Anthony Infantolino, Mitchell Conn

**Affiliations:** ^1^Department of Medicine, Thomas Jefferson University Hospital, 132 S 10th Street, Philadelphia, PA 19107, USA; ^2^Division of Gastroenterology and Hepatology, Department of Medicine, Thomas Jefferson University Hospital, 132 S 10th Street, Philadelphia, PA 19107, USA

## Abstract

*Background and Aim*. Spiral enteroscopy (SE) is a new small bowel endoscopic technique. Our aim is to review the diagnostic and therapeutic yield, safety of SE, and the predictive role of prior capsule endoscopy (CE) at an academic center. *Methods*. A retrospective review of patients undergoing SE after prior CE between 2008 and 2013 was performed. Capsule location index (CLI) was defined as the fraction of total small bowel transit time when the lesion was seen on CE. *Results*. A total of 174 SEs were performed: antegrade (147) and retrograde (27). Abnormalities on SE were detected in 65% patients. The procedure was safe in patients with surgically altered bowel anatomy (*n* = 12). The diagnostic yield of antegrade SE decreased with increasing CLI range. The diagnostic yield of retrograde SE decreased on decreasing CLI range. A CLI cutoff of 0.6 was derived that determined the initial route of SE. Vascular ectasias seen on CE were detected in 83% cases on SE; *p* < 0.01. *Conclusions*. SE is safe with a high diagnostic and therapeutic yield. CLI is predictive of the success of SE and determines the best route of SE. The type of small bowel pathology targeted by SE may affect its utility and yield.

## 1. Introduction

The advent of wireless capsule endoscopy has remarkably improved the endoscopic evaluation of the small bowel [1–3]. However, one of the major limitations of capsule endoscopy is that it is a purely diagnostic procedure. The introduction of double balloon enteroscopy in 2001, followed by single balloon enteroscopy, has allowed for the ability to perform diagnostic and therapeutic procedures deep into the small bowel [4–7]. More recently, spiral enteroscopy (SE) has been introduced for deep intubation of the small bowel. Double balloon enteroscopy and single balloon enteroscopy use a similar mechanism of advancement by sequential bowel pleating by a push-pull technique. In contrast, SE uses a plastic spiral tipped overtube coupled with an enteroscope that can be advanced as a unit by continuous rotation of the overtube. SE has not been studied in a large series and is still an emerging technique. Our institution has one of the largest experiences with this technique.

The most common indication for deep enteroscopy is small bowel pathology detected on prior capsule endoscopy. It has been a challenge to accurately detect lesions seen on capsule endoscopy via deep enteroscopy. Although SE has been initially shown as a safe and feasible means of deep small bowel intubation, its ability to detect lesions seen on capsule endoscopy has not been well reported. The time taken by the video capsule to traverse the small bowel varies from minutes to hours and may vary in different patients. This variation makes it difficult to predict the possible location of the lesion in the small bowel as well as the route most suitable for enteroscopy (antegrade or retrograde). Insertion of enteroscope via mouth is called antegrade approach while insertion of enteroscope via anus is called retrograde approach. We calculated an index called a capsule location index (CLI) that gives the location of the lesion in terms of the fraction of time of small bowel transit.

One of the aims of our study was to assess the safety as well as diagnostic and therapeutic yield of SE using either an antegrade or retrograde approach. In addition, we sought to determine if CLI is predictive of the success of SE and can help determine the best route of enteroscopy.

## 2. Materials and Methods

A retrospective analysis of patients who underwent capsule endoscopy and subsequent SE at Thomas Jefferson University Hospital between 2008 and 2013 was performed. The institutional review board of Thomas Jefferson University approved the study. The patients' electronic medical records, capsule endoscopy studies, and spiral enteroscopy reports were reviewed.

### 2.1. Capsule Study

**Figure 1:**
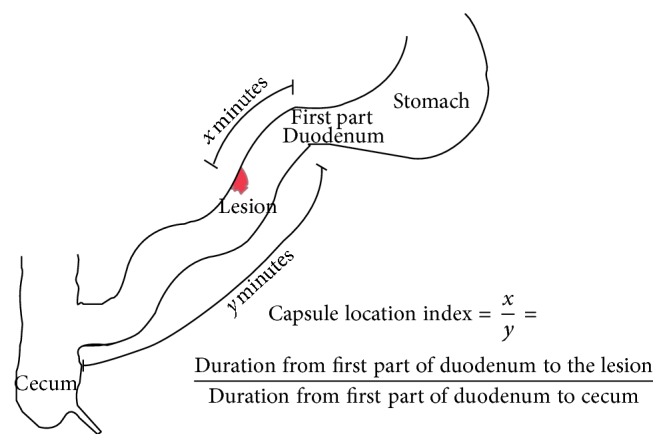
Schematic diagram for the calculation of Capsule Location Index.

The PillCam SB (Given Imaging, USA) was used in all patients in this study. Patients underwent capsule endoscopy after a 12-hour fast with clear liquids allowed up to 4 hours before the test. Capsule endoscopy was evaluated by only one gastroenterologist expert in viewing capsule studies. Capsule endoscopy was considered positive if any lesion was detected in the small bowel. If two different lesions were seen, both the lesions were recorded and considered as two separate target lesions on spiral enteroscopy. The following information was recorded: the type of small bowel lesion found, time at the first duodenal image, time at which lesion is seen, time to reach the cecum, and small bowel transit time. CLI was derived to describe the location of the lesion as a time fraction of small bowel transit (Figure 1). It was calculated as the fraction of total small bowel transit time when the lesion was seen. Total small bowel transit time was defined as the time interval between the first duodenal image and the first cecal image:

(1) CLI=Duration from the first duodenal image to the lesion foundDuration from the first duodenal image to cecum

Not all the capsule endoscopies were complete to reach the cecum. Incomplete capsule endoscopies were defined as capsule not reaching the cecum within the recorded time. CLI could not be calculated in those studies.

### 2.2. Spiral Enteroscopy

A total of 174 SE procedures were performed in 148 patients. 19 patients had the procedure more than once either for inability to detect lesions or for persistent symptoms. Nine patients out of 19 had both antegrade and retrograde spiral procedures. For the sake of uniformity, each procedure was considered to be performed on a unique patient as the major outcome was to study the detection of lesion. Ninety-six percent (168/174) of all spiral enteroscopy procedures were performed by only one expert endoscopist who has been performing all forms of deep small bowel enteroscopy since 1990. The remaining six spiral enteroscopy procedures were performed by another expert endoscopist having expertise in double balloon enteroscopy. All procedures were carried out with the Olympus SIF-Q180 enteroscope (Spiral Medical, LLC, MA, USA) coupled with a spiral overtube. General anesthesia was used for all antegrade procedures and deep sedation was used for retrograde cases. Spiral enteroscopy was performed as previously described [8]. Examination of the small bowel and therapeutic maneuvers were performed during intubation and withdrawal of the enteroscope and overtube. The patients were followed up for any complications till discharge from the endoscopy unit as well as the next day via telephone call or inpatient follow-up. They were also asked to call the physician for any new symptoms.

Positive findings on SE were compared to the abnormal findings seen on capsule endoscopy. Studies in which SE identified the lesion seen on capsule endoscopy were considered positive. In cases where two different types of lesions were seen on capsule endoscopy, both the lesions were considered to be target lesions for SE. For cases in which multiple vascular ectasias were the target lesions on capsule endoscopy, detection by SE was considered positive if any vascular ectasias were detected on SE.

Inclusion criteria included patients of all ages who required deep enteroscopy for further evaluation of the lesions detected on capsule endoscopy, computed tomography (CT) scan, or CT enterography.

Exclusion criteria included patients with severe comorbidities precluding sedation and general anesthesia as well as patients with known esophageal varices and strictures precluding overtube insertion.

Data were recorded in an electronic database. Statistical analysis was performed using the SAS system, version 9.1 (SAS Institute Inc., Cary, NC). Chi-square test and Fisher's exact test were used for categorical variables. A *p* value of <0.05 was considered statistically significant.

## 3. Results

A total of 174 SE procedures were performed between 2008 and 2013. 147 were antegrade and 27 were retrograde SE. Mean age of the patients was 66 (±14.6) years. 46% (81) were females and 54% (93) were males.

### 3.1. Indications of SE

**Figure 2:**
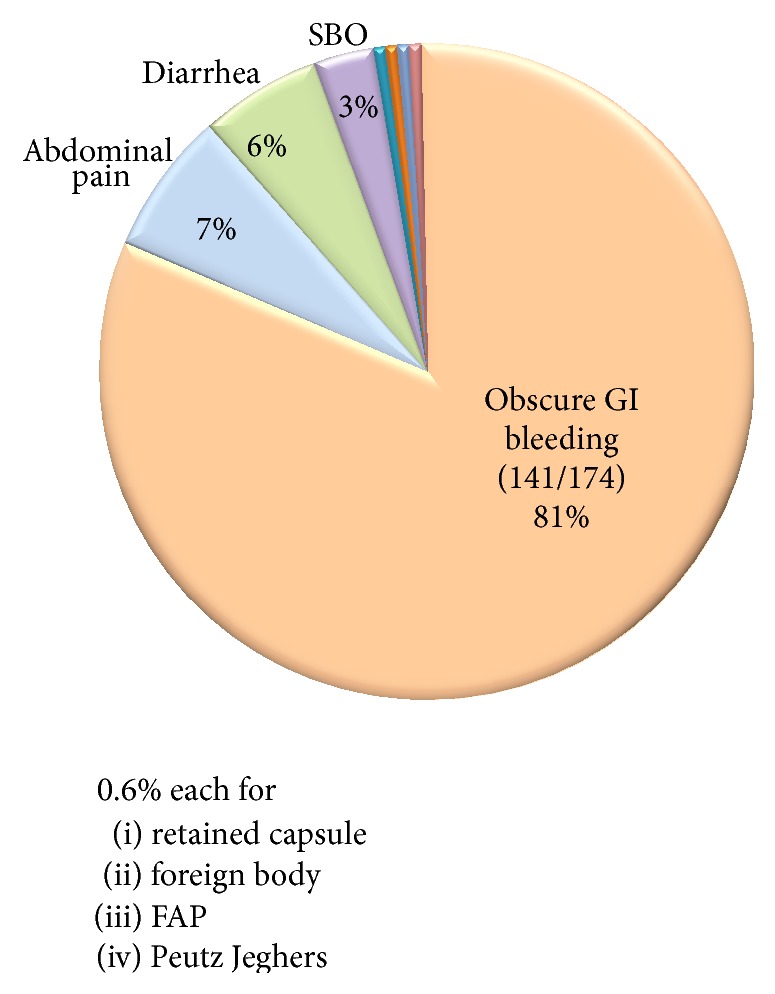
Overall indications of SE.

The most common indication of SE was obscure GI bleeding (141, 81%).  Figure 2 shows various indications of SE. Most SEs were performed due to abnormal prior capsule endoscopy.  Table 1 provides the indications for SE based on the abnormality seen on capsule endoscopy. Vascular ectasia seen on the capsule endoscopy was the most common indication for SE with inflammation being the least common.

### 3.2. Yield of SE

SE yielded positive findings in 113/174 (65%) patients.  Table 2 shows the various findings revealed on spiral enteroscopy. Vascular ectasias were the most common finding (60%). There were 5 patients with Dieulafoy's lesion in the small bowel. Two patients showed active jejunal diverticular bleeding.

### 3.3. Therapeutic Yield of SE

Endoscopic therapy was performed in 63% (109) of patients (Table 3). The most common therapy performed was argon plasma coagulation (APC), performed in 61% (66) of patients. Endoscopic clipping was successfully performed in 20 patients. Dilation of strictures as well as retrieval of endocapsule was also performed with SE.

### 3.4. Safety of SE

SE was safe in patients with a history of surgically altered bowel anatomy. It was safe in seven patients having Roux-en-Y bypass and five patients having enteroenteral anastomosis. There were no major complications in all 174 patients. Four patients had minor complications. There were three patients with esophageal mucosal tears who were successfully treated conservatively or endoscopically with endoclips, while one patient had transient jejunal intussusception that resolved spontaneously during the procedure.

### 3.5. SE in relation to Prior Capsule Endoscopy

There were 132 prior positive capsule studies available to review. 115 out of 132 studies were complete (capsule reached the cecum within the recorded time) to calculate CLI.

The median time interval between capsule endoscopy and SE was one month. For active bleeding seen on capsule endoscopy, SE was performed within next 24 hours.

**Figure 3:**
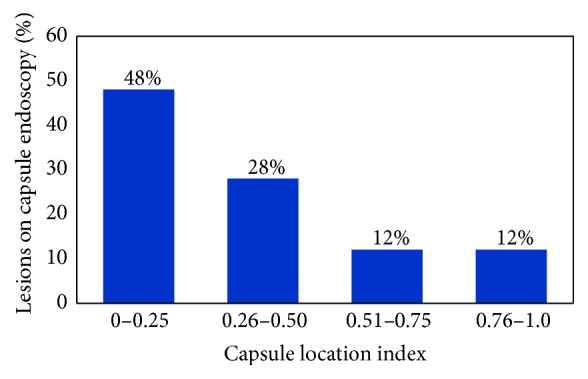
Distribution of lesions seen on capsule endoscopy with respect to capsule location index (CLI).

Almost half of the lesions were present in the first quarter of the small bowel transit time (0–0.25) (Figure 3).

### 3.6. Antegrade SE

**Figure 4:**
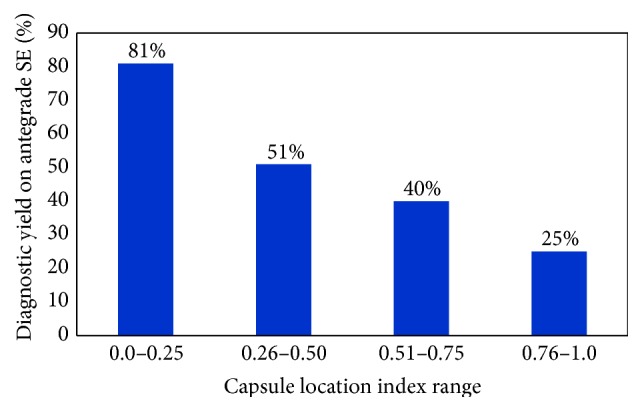
The diagnostic yield of antegrade SE with respect to CLI. Note that the yield decreases as the CLI range increases.

The detection rate of lesions seen on complete capsule endoscopy by antegrade approach was 64.9% (76/117 lesions). Mean CLI was 0.22 in positive antegrade SE with a range of 0.00 to 0.72, while a negative antegrade enteroscopy had a mean CLI of 0.40 with a range of 0.00 to 0.86. Figure 3 illustrates the decreasing diagnostic yield of antegrade SE at increasing range of CLI. There was significant difference in the diagnostic yield for lesion within CLI range (0.0–0.25), 81% (51/63) as compared to lesions within CLI range (0.26–1.0) 46% (25/54) (*p* < 0.001) (Figure 4).

### 3.7. Retrograde SE

**Figure 5:**
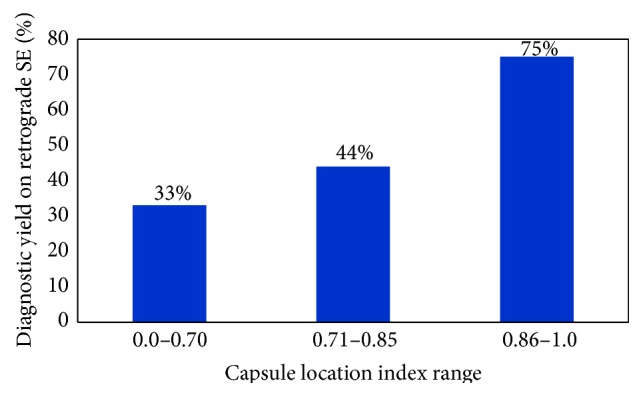
The diagnostic yield of retrograde SE with respect to CLI. Note that the yield decreases as the CLI range decreases.

The detection rate of lesion seen on capsule endoscopy by retrograde approach was 55% (11/20 lesions). Mean CLI was 0.88 for positive retrograde enteroscopy while negative retrograde enteroscopy had a mean CLI of 0.76. As shown in Figure 5, the diagnostic yield of retrograde SE decreases as the range of CLI decreases.

### 3.8. CLI Cut Offs

We sought to determine the capsule location index cutoffs in order to achieve a diagnostic yield of at least 70% in antegrade as well as retrograde SE, respectively, to be able to decide the initial route of enteroscopy.

**Figure 6:**
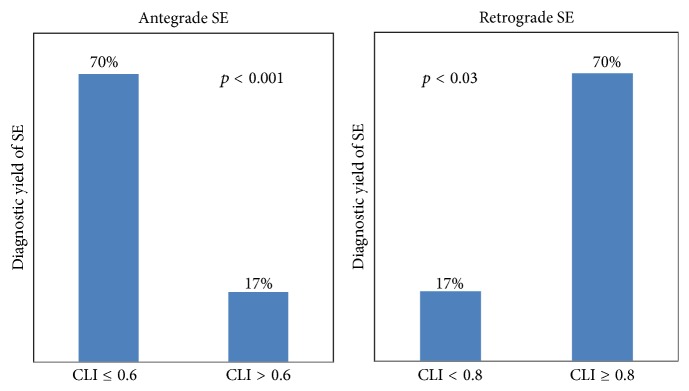
CLI cut offs for achieving 70% diagnostic yield on antegrade as well as retrograde approaches respectively.

Lesions within CLI of 0.6 were detected 70% of the times by initial antegrade approach. Diagnostic yield of 70% was achieved for CLI up to 0.6 versus 17% for lesions with CLI > 0.60, *p* < 0.001 (Fisher's test). Similarly, lesions with CLI ≥ 0.80 were detected 70% of the times by initial retrograde approach. There was diagnostic yield of 70% with lesions with CLI ≥ 0.80 versus 17% with CLI < 0.80, *p* = 0.03 (Fisher's test) (Figure 6). A CLI range of 0.61 to 0.79 had the lowest yield of 15% (2/13) by either approach (9 antegrade and 4 retrograde).

### 3.9. Yield of SE with respect to Various Lesions on CE

SE was able to detect lesions seen on capsule study at various rates based on the type of lesion (Table 4). Vascular ectasias seen on capsule study had the highest detection rate on spiral enteroscopy (83%), while mass/bulging had the lowest detection rate (32%). Active bleeding seen on capsule study was detected in 64% of cases with spiral enteroscopy. The overall detection rate of SE after capsule endoscopy was 62% (81/131).

## 4. Discussion

With the advent of capsule endoscopy, direct and painless evaluation of the entire small bowel became possible. Capsule endoscopy is considered to be superior to angiography, small bowel follow-through, and conventional push enteroscopy in detection of small bowel disease pathology especially in patients with bleeding [9]. However, one of the major limitations of capsule endoscopy is that it is a purely diagnostic procedure [10]. With the introduction of deep enteroscopy it is now possible to identify and treat lesions detected on capsule endoscopy. Spiral enteroscopy is the newest technique for deep small bowel intubation. Our institution has one of the largest experiences with SE. Preliminary data have shown SE to be a safe and well tolerated procedure [11].

Our results indicate that SE is a safe and effective deep enteroscopic technique. The diagnostic and therapeutic yields of the SE procedures in this study compare favorably with data previously published regarding balloon enteroscopy [12, 13]. The yield is also consistent with the recent preliminary data on SE [14]. In our study the most common indication for SE was obscure gastrointestinal bleeding and the most common finding was vascular ectasias (VE). It allows for endoscopic therapy such as argon plasma coagulation, clipping, biopsy, polypectomy, dilation of stricture, and injection of solution. We also successfully treated an active jejunal diverticular bleeding [15].

The procedure was well tolerated by the majority of the patients. It appears to be safe and therapeutic in patients with Roux-en-Y bypass and enteroenteral anastomosis. No major complications were noted. Esophageal mucosal tear occurred during withdrawal of the scope in three patients (2%). They were successfully treated. The utility of SE may be limited in patients with known esophageal pathology. The spiral enteroscopy technique may have an advantage over other deep enteroscopy techniques in terms of speed of advancement and stability while doing maneuvers and during withdrawal [11]. Endoscopic therapeutic procedures are easy to perform during SE. The configuration of the enteroscope and the overtube remains relatively straight, without significant loop formation which permits easy passage of the accessories [11]. In a study by Akerman et al., the rapidity of advancement of the enteroscope during spiral enteroscopy was superior to that of other deep small bowel intubation techniques with similar estimated depths of insertion [11].

SE like other deep enteroscopies may require both an antegrade and a retrograde study to investigate whole small bowel. The ability to select the best approach based on prior capsule endoscopy findings can improve success and minimize the need of multiple procedures and additional sedation. In a recent study evaluating the insertion route of push or pull enteroscopy, Gay used a % time index which was defined as lesion location as a percentage of the mouth to cecum time [16]. However, gastric emptying time varies in patients and we found that time to reach duodenum varies frequently. Li et al. used pylorus to cecum time to determine lesion location [10]. However, since pylorus is a sphincter, it may affect the passage of capsule. Since duodenum and cecum are stable landmarks we used small bowel transit time from the first part of duodenum to cecum to determine location of the lesion.

In our study, an antegrade approach CLI cutoff ≤ 0.25 revealed the highest yield of 81% and only a yield of 46% for the lesions with CLI > 0.25. CLI within 0.6 had a 70% diagnostic yield with initial antegrade approach. Initial antegrade approach should be considered for lesions with CLI up to 0.6. For retrograde approach, a CLI of ≥0.8 had a diagnostic yield of 70%. Initial retrograde approach should be considered for lesions with CLI ≥0.8. Lesions with CLI between 0.61 and 0.79 remain a challenge, as they were successfully detected only 15% of the time by either approach. This may guide us in informing the patients about the low likelihood of detection of a lesion before going for long standing enteroscopy procedure requiring general anesthesia. They may require a more invasive/surgical procedure.

For those cases in which capsule endoscopy is incomplete, antegrade SE should be the first procedure of choice if a pathologic lesion was seen on capsule endoscopy. A repeat capsule endoscopy could be considered with or without endoscope placement if gastric emptying time contributed to the incomplete study.

Our study has somewhat similar results to the study by Li et al. which utilized double balloon enteroscopy. Some minor differences are as follows: the cutoff of retrograde approach derived in their study was 0.6 compared to 0.8 in our study. We believe that, for lesions with CLI between 0.6 and 0.8, initial approach should be individualized based on the presentation of the patient and informing the patient about the low chances of lesion to be detected by either approach. The prior study excluded patients with positive capsule studies showing submucosal tumor but with subsequent negative balloon enteroscopy via either approach which may affect the yield of the enteroscopy procedure. In comparison, we included all the patients with positive capsule endoscopy with subsequent negative spiral enteroscopy. We included 22 capsule studies showing submucosal tumor, with lowest yield on spiral enteroscopy (7/22, 32%). It is important to note that there is a false positive rate for capsule endoscopy which is difficult to confirm. One other note is that there were higher cases of obscure gastrointestinal bleeding in our study compared to Li et al. study (81% versus 67%). Since bleeding is often self-limited and the lesion may heal in time, it might have led to some differences in the diagnostic yield.

Vascular ectasias were the most common finding on capsule endoscopy and SE. 83% of the vascular ectasias seen by capsule endoscopy were reached by SE. Similarly, active bleeding was also easily detected on SE (64% of studies). This shows that capsule endoscopy may be indicated as an initial test in patients with obscure active gastrointestinal bleeding followed by SE, as angiographic embolization may not be a preferred option in small bowel bleeding source due to risk of ischemia.

Other findings such as ulcer, mass/bulging, polyp, and inflammation often are single and may be missed during enteroscopy. These lesions may also be nonspecific, transient, and overdiagnosed during capsule endoscopy. One factor may be the time interval between capsule study and enteroscopy. Most of the cases in our study had a median interval of 1 month. It may be possible that lesions such as ulcer or inflammation may have healed over time. This delay may lead to lower detection rate of these types of lesions on spiral enteroscopy.

In a recent study, Buscaglia et al. evaluated the diagnostic yield of SE performed for evaluation of abnormal capsule findings on smaller group of patients (56 patients) and included only antegrade approach. Findings on capsule study were detected by SE in 30/56 (53.6%) cases, comparable to our detection rate of 62% [17]. Their main focus was to determine the yield of SE and not to predict the route of enteroscopy or success of enteroscopy.

The strengths of the study are as follows. (1) There are a large number of patients compared to previous two studies [10, 17]. (2) Almost all the procedures were performed by a single expert endoscopist. This decreases the variability in performance and detection rates. The major limitation of the study is its retrospective nature. Also as mentioned earlier, the time interval between capsule endoscopy and spiral enteroscopy may affect the detection rate of the lesions by spiral enteroscopy.

In conclusion from this large volume retrospective study, SE is safe with a high diagnostic and therapeutic yield. CLI is predictive of the success of SE and determines the best route of SE. Lesions within a location index of 0.6 should be approached first with antegrade route while those with a location index ≥ 0.8 should be initially approached by retrograde route. The type of small bowel pathology targeted by SE may affect its utility and yield.

## Figures and Tables

**Table 1:** Indications for SE based on capsule endoscopy findings.

Findings on capsule endoscopy	*N* (number of patients)
Vascular ectasia (VE)	35
Active bleeding	25
Mass/bulging	22
Blood	19
Ulcer	12
Polyp	11
Inflammation	8

*Note*. There were 16 patients with obscure GI bleeding who had prior capsule endoscopy not available for review.

**Table 2:** Findings on SE.

Findings	*N* (number of cases)
Vascular ectasia (VE)	60 (35%)
Inflammation	19 (11%)
Polyp	7 (4%)
Ulcer	7 (4%)
Mass	7 (4%)
Benign small bowel diverticula	6 (3.4%)
Dieulafoy's lesion	5 (2.8%)
Scalloping of the mucosa	5 (2.8%)
Small bowel diverticular bleeding	2 (1.1%)
Active bleeding of unknown cause	2 (1.1%)
Stricture	2 (1.1%)
Small bowel varices	1 (0.6%)

Ten patients had two findings on spiral enteroscopy.

**Table 3:** Various therapeutic maneuvers performed by SE.

Therapy performed	*N* (number of cases)
APC	66 (38%)
Biopsy	53 (31%)
Endoclip	20 (11.5%)
Polypectomy	7 (4%)
Dilatation of stricture	2 (1.1%)
Retrieval of endocapsule	1 (0.6%)

**Table 4:** Detection rate of various lesions seen on capsule endoscopy by SE.

	Lesion on capsule	Corresponding SE	Detection rate
Vascular ectasia (VE)	35	29	83%
Blood	19	13	68%
Active bleeding	25	16	64%
Ulcer	12	7	58%
Inflammation	8	4	50%
Polyp	11	5	45%
Mass/bulging	22	7	32%
